# Maternity care experience of Pakistani ethnic minority women in Hong Kong

**DOI:** 10.3389/fpubh.2023.1009214

**Published:** 2023-03-01

**Authors:** Saba Asim, Elena Nichini, William Bernard Goggins, Dong Dong, Eng-King Yeoh

**Affiliations:** ^1^JC School of Public Health and Primary Care, The Chinese University of Hong Kong, Shatin, Hong Kong SAR, China; ^2^Centre for Health Systems and Policy Research, JC School of Public Health and Primary Care, Hong Kong, Hong Kong SAR, China

**Keywords:** Pakistani women, maternity care, ethnic minority, immigrant, Hong Kong

## Abstract

**Background:**

Persistent inequalities in maternity care experience and outcomes exist globally for ethnic minority (EM) and migrant women. Despite the fact that this is an important research area, no prior study has been done in Hong Kong (HK) to examine maternity care experience of EM women.

**Objectives:**

To investigate maternity care experience of Pakistani EM women (both local born and immigrants) during pregnancy, birth and after birth in hospital in HK. An evaluation of their satisfaction and factors predicting satisfaction with care during the three phases of maternity care was included in the study.

**Methods:**

A cross sectional survey was conducted among Pakistani EM women who had given birth in HK in last 3 years, using a structured questionnaire by a bilingual interviewer, from April to May 2020. Counts and percentages were used to describe all categorical variables. Association between predictor variables and overall satisfaction was assessed by bivariate analysis and multiple logistic regression.

**Results:**

One hundred and twenty questionnaires were completed. Almost 60 percent of the women were very satisfied with the overall care. More than half of the women described the care they received as kind, respectful and well communicated. After adjusting for age and parity, HK born Pakistani women expressed relatively less satisfaction with care, especially during pregnancy and labor and birth, as compared with Pakistan born women. Women with conversational or fluent English-speaking ability also felt comparatively less satisfied particularly from intrapartum and postnatal care in hospital. Education level had a negative association with satisfaction with care during pregnancy.

**Conclusions:**

Maternity care providers should take into account the diversity of EM women population in HK. Our findings suggest that effective communication and care that can meet individual needs, expectations, and values is imperative to improve experience and quality of maternity care for EM women in HK.

## 1. Introduction

Maternity care, which is the healthcare received throughout pregnancy, during labor and childbirth and up to 6 weeks after childbirth, is of vital significance for maternal and child health. As such, policies have been implemented worldwide to ensure provision of equitable good quality maternity care to every pregnant woman and child. Such efforts incorporated the agenda of the United Nations Millennium Development Goals 4 and 5 (MDG, 2000–2015) and later the Sustainable Development Goal 3 (SDG, 2016–2030) (1, 2). In articulation with this, the World Health Organization (WHO) also formulated the new Global Strategy for Women's, Children's and Adolescents' Health (2016–2030) for the SDG era in conjunction with the global community (3).

Despite these efforts, persistent inequalities in maternity care experience and outcomes exist globally, particularly for ethnic minority and migrant women (4–6). These women tend to have higher susceptibility to anxiety and depression as compared to majority and native women (7, 8). Moreover, there is potentially increased risk of prematurity, low birth weight, increased maternal and infant morbidity and mortality (9, 10). Previous research identified several factors shaping these inequalities, including communication gap and lack of information due to language and cultural differences, structural barriers, stereotyping, discrimination and social exclusion (11, 12). Other factors included socio-economic status, poverty, gender inequalities and living in highly deprived areas (10, 12).

While branding itself as an international and inclusive city, Hong Kong holds a hegemonic Chinese culture where discrimination and marginalization of ethnic and migrant communities are widespread (13). Despite their deep-rooted presence in the city since its British colonial times, South Asians in general, and the Pakistani community in particular, is one of the most marginalized EM population in HK in terms of economic, social and educational levels (14). Such marginalization is also reflected in the health sector where they are exposed to higher health risks yet poorer access to healthcare services (15, 16); women in particular felt disengaged with the HK healthcare system (17).

Such marginality is likely to have detrimental effects on maternity care experience of Pakistani EM women in HK. Research conducted in Western countries has in fact documented adverse perinatal outcomes and poor maternity care experience of Pakistani EM women. A study from the United Kingdom (UK) for instance investigated increased perinatal mortality in Pakistani EM women and found that they had more risk factors, including low birth weight, diabetes, gestational diabetes, less BMI (<18 kg/m2), high parity and delayed bookings (>12 weeks) (18). Another UK study indicated that very few Pakistani mothers took pre-conception folic acid as they were less aware of the role of folic acid in congenital anomalies prevention (19). Low utilization of pain relief during birth was more common among Pakistani women, due to low education, less native language speaking ability and discrepancies in information availability. As a result, these women felt unsupported and dissatisfied with the maternity care they received (20–22). Conversely, Pakistani women with higher education were highly confident, well supported and informed appropriately (20).

Although HK has one of the lowest maternal mortality and infant mortality rate in the world (23), yet these indicators alone are insufficient in measuring quality care. WHO maternal and new-born quality care framework emphasized on experience of care as a key domain along with clinical care provision and availability of human and physical resources (24). Women satisfaction with care is a quality indicator to measure experience of care that should be evaluated for identifying areas for action to provide good quality maternity care (25). The objective of this study is to investigate maternity care experience of Pakistani EM women (both local born and immigrants) during pregnancy, birth and after birth in hospital in HK. An evaluation of their satisfaction and factors predicting satisfaction with care during the three phases of maternity care was also included in the study.

## 2. Materials and methods

### 2.1. Study design, setting, and period

A cross-sectional study was conducted in HK from April to May 2020. Due to the social distancing measures during COVID-19, the survey was done on phone through WhatsApp video call. Ethics approval was obtained from the Survey and Behavioral Research Ethics Committee of the University (SBRE-19-521).

### 2.2. Populations and eligibility

All women living in HK, aged 18 and above, self-identified themselves as Pakistani and had given birth to at least one child in HK in the past 3 years, were included as the target population of the study. Women whose baby had died were excluded.

### 2.3. Sample size determination and sampling procedures

Purposive and snowball sampling was employed to recruit the eligible participants. The participants were reached through local Pakistani WhatsApp groups, social networks, non-governmental organizations working for ethnic minorities and community workers. The purpose and procedure of the study was explained to them and those who agreed to join were contacted to set the schedule for the interview.

Sample size was estimated by assuming 50% women satisfaction with maternity care services and 95% confidence interval with 7% error of margin. It was calculated by the equation (26):


$$\begin{array}{l} N=\frac{\left[z_{\frac{\alpha }{2}}^{2}\times \;p\times \left(1-p\right)\times DEFF\right]}{d^{2}} \end{array}$$

Where α (Probability of type 1 error) = 0.05, p (Prevalence proportion) = 0.5, DEFF (Effect Size) = 1, d (Absolute precision) = 0.07, N (Sample size) = 196. We planned to interview 200 Pakistani women.

### 2.4. Data collection tools and procedures

Data was collected through a structured, interviewer administered paper-based questionnaire, adopted from the Survey of Bangladeshi women's experience of maternity services (SBWEMS) (27). After obtaining the informed consent, the participants were interviewed to fill the questionnaire on phone through WhatsApp video call at the prescheduled date and time by a bilingual interviewer. The interview was conducted in English or Urdu depending upon participant's preference. On an average the interview lasted for 45–60 min.

### 2.5. Outcome variables and measurements

The primary outcome variable was women's overall satisfaction with care in each of the three phases of pregnancy. For overall satisfaction participants were asked “Thinking back now, how satisfied are you, overall, with the care you received during your pregnancy, labor and delivery and after birth (in separate questions)?” Responses were recorded on a 4 point Likert scale: very satisfied, somewhat satisfied, somewhat dissatisfied and very dissatisfied. For statistical analysis, the answers were dichotomized into “high satisfaction (Very satisfied)” and “less satisfaction (somewhat satisfied, somewhat dissatisfied, and very dissatisfied)”. This was done firstly because not many women opted for the somewhat dissatisfied or very dissatisfied categories. Secondly from previous research we found that a rating of “very satisfied” is used as indicator for optimal care with lower ratings pointing out that there was room for improvement (28, 29). Further maternity care experience was assessed in terms of access to care, information exchange and perceptions of care. These were evaluated by questions regarding choice of gender of health care professionals, continuity of care, interactions with staff, availability of interpreters, information leaflets, and satisfaction with analgesia, length of stay in the hospital and preferences for care.

### 2.6. Data quality control

We employed the SBWEMS questionnaire which had good validity and reliability in examining ethnic minority women's satisfaction with antenatal, intrapartum and postnatal care (27). The questionnaire was pilot tested with 10 Pakistani EM women to check any inconsistencies and objections and revised accordingly.

### 2.7. Data processing and analysis

All the data was checked for completeness and entered in SPSS version 24.0, which was also used for all analysis. Counts and percentages were used to describe all categorical variables including the variables related to outcome variables. Bivariate analyses, including the chi-square test, Fisher's exact test was performed to test the associations between individual predictors and the outcome variables (not shown in results). Multiple logistic regression analysis was then done with the significant independent variables forthree outcome (dependent) variables: less satisfaction with care during pregnancy, labor and birth and after birth. *P*-value < 0.05 was considered statistically significant. Age and parity were adjusted as confounders. The fit of the model was tested using the Hosmer-Lemeshow test.

## 3. Results

One hundred and twenty questionnaires were completed. Table 1 shows the profile of the participants. The majority of the women were Pakistan born (81.7%), aged at 34 years and below (80.0%), had delivered vaginally (71.7%), at term (82.5%) and had normal birth weight babies (83.3%). About two-thirds of them were multiparous (69.2%) and had education level of Secondary or below (65%). Half of the respondents spoke Urdu as their primary language followed by other regional languages of Pakistan, whereas English and Cantonese were less often spoken as primary languages. Table 2 shows the self-described language ability of the respondents. More than half (60%) were able to speak and two-thirds could read, write and understand English either well or fluently. Only 23.3 and 21.6% women could understand and speak Cantonese well or fluent and very few could read or write.

**Table 1:** Characteristics of study participants.

**Characteristics**	***N =* 120 *n* (%)**
**Age group (yrs.)**	
15–24	17 (14.2)
25–34	79 (65.8)
36 and above	24 (20.0)
**Parity**	
Prim parous	37 (30.8)
Multiparous	83 (69.2)
**Gestation at birth**	
≥37 weeks	99 (82.5)
<37 weeks	21 (17.5)
**Infant birth weight**	
≥2.51 kg	100 (83.3)
< 2.5 kg	20 (16.7)
**Type of delivery**	
Vaginal delivery	83 (69.2)
Vaginal assisted by equipment	03 (2.5)
A planned cesarean delivery	16 (13.3)
An emergency cesarean delivery	18 (15)
**Country of birth**	
Pakistan	98 (81.7)
Hong Kong	21 (17.5)
Others	01 (0.8)
**Education level**	
Secondary and less	78 (65)
Some university and above	42 (35)
**Primary language**	
Urdu	60 (50)
English	2 (1.7)
Cantonese	1 (0.8)
Others	57 (47.5)

**Table 2:** Language ability of the participants.

**Languages**		**Not at all *n* (%)**	**With difficulty *n* (%)**	**Well *n* (%)**	**Fluent *n* (%)**
Urdu	Speak	0	6 (5)	53 (44.2)	61 (50.8)
	Read	19 (15.8)	11 (9.2)	41 (34.2)	49 (40.8)
	Write	24 (20)	13 (10.8)	36 (30)	47 (39.2)
	Understand	0	5 (4.2)	56 (46.7)	59 (49.2)
English	Speak	14 (11.7)	34 (28.3)	39 (32.5)	33 (27.5)
	Read	19 (15.8)	18 (15)	40 (33.3)	43 (35.8)
	Write	20 (16.7)	19 (15.8)	38 (31.7)	43 (35.8)
	Understand	12 (10)	27 (22.5)	38 (31.7)	43 (35.8)
Cantonese	Speak	66 (55)	28 (23.3)	19 (15.8)	7 (5.8)
	Read	100 (83.3)	11 (9.2)	7 (5.8)	2 (1.7)
	Write	101 (84.2)	8 (6.7)	9 (7.5)	2 (1.7)
	Understand	60 (50)	32 (26.7)	21 (17.5)	7 (5.8)

### 3.1. Antenatal care

During the antenatal care (Table 3), more than half (57.6%) of the women had choice of time of antenatal checkups but only 22% of the women were given choice of whether to have a man or woman perform their antenatal checkups. Two-thirds (67%) of the women strongly agreed that they were always encouraged to ask questions and always understood the advice given during their checkups. Almost two-thirds (63.3%) had enough time talking to doctors or midwives. Twenty-two percent of the women were provided with information leaflets in their primary language and 59% of those who received them reported that they were understandable and comprehensive. Majority of the women were provided interpreters by HCPs (73.8%). Only ten women attended antenatal classes and 61.7% did not attend the classes due to their lack of knowledge of the existence of these classes.

**Table 3:** Access to care, information exchange and perceptions of care during the three phases of maternity.

**During pregnancy**	
Have choice of time of antenatal check-ups	68 (57.6)^†^
Have choice with whom to have antenatal checkups (man or woman)	26 (22.0)^†^
Received continuous antenatal care from one or two people or different of the same team	30 (25)
Attended antenatal classes	10 (8.3)
Did not know about antenatal classes	74 (61.7)
Received nutritional supplements (folate, iron, calcium)	82 (68.3)
Provided information leaflets in primary language	27 (22.5)
Professional interpreter provided by the Healthcare professionals	31 (73.8)^‡^
Explained enough information about tests and procedures needed	94 (78.3)
Had enough time talking to doctors or midwives during check-ups	76 (63.3)
Always encouraged to ask questions and understood advice given	80 (66.7)
Never felt treated differently to other people by HCP	96 (80)
**During labor and birth**	
Choice about type of healthcare professional (man or woman)	31 (25.8)
During labor and birth allowed to have choice of support people	87 (72.5)
Allowed to move around during labor	37 (30.8)
Right number of hospital staff around during labor and birth	92 (76.7)
Needed interpreter	31 (25.8)
Was aware of the availability of professional interpreter	29 (93.5)^§^
Had someone to provide interpreting services	26 (83.9)^§^
Offered choice of interpreter by the HCP	13 (41.9)^§^
Very satisfied with the communication with HCP	74 (61.7)
Had met staff before	26 (21.7)
HCP treated with respect for most of the time	73 (60.8)
Doctors spent enough time during labor and birth	73 (60.8)
Midwives spent enough time during labor and birth	88 (73.3)
Very satisfied with the pain relief received	57 (47.5)
Left alone and worried in labor	25 (20.8)
Left alone and worried after birth	22 (18.3)
Staff looked after in a very kind way during labor and delivery	61 (50.8)
Never felt treated differently to other people by HCP	89 (74.2)
**After birth in hospital**	
Postnatal stay 3 days or more	80 (66.7)
Felt length of stay about right	97 (80.8)
Baby had skin to skin contact in the first hour after birth	84 (70)
Offered to help start breastfeeding in first hour after birth by the HCP	37 (30.8)
Baby was exclusively breast milk fed vs. both breast and formula milk or only formula milk in the first few days	41 (34.2)
Received enough breast feeding advice and support	100 (83.3)
Always understood the information provided	70 (58.3)
Felt got enough help with own needs from hospital staff	100 (83.3)
Felt got enough help with baby from hospital staff	99 (82.5)
Happy with visiting times	79 (65.8)
Staff looked after in a very kind way following the birth of baby	68 (56.7)
Strongly agree doctors spent enough time	43 (35.8)
Strongly agree midwives spent enough time	59 (49.2)
HCP asked if had any preference or wanted to follow any particular custom after birth of baby	11 (9.2)
HCP asked about any food preference	105 (87.5)

Counts with percentages, N = 120.

† Total number of participants = 118, 02 women did not receive initial antenatal care in Hong Kong.

‡ Total number of participants = 42, rest did not require interpreting services.

§ Total number of participants = 31, rest did not require interpreting services.

### 3.2. Intrapartum care

Regarding care during labor and birth (Table 3), most women (73%) were allowed to have choice of supporting people during labor and birth. The majority (84%) had someone to provide interpreting services during labor and birth and of these 42% of the women were offered choice of interpreter by the HCPs. Less than two thirds (61.7%) were very satisfied with the communication with HCP and only half (50.8%) reported a very kind attitude of staff during labor and birth. Less than half (47.5%) of the women were very satisfied with the pain relief received during labor and only 31% were allowed to move during labor.

### 3.3. Postnatal care in hospital

With respect to care during postnatal period in hospital (Table 3), most of the women (70%) were given skin to skin contact with baby in the first hour after birth. However, only 31% were offered the chance to start breast feeding in the first hour after birth, only 34% of the babies were exclusively breast fed and 40% of the babies received both breast and bottle milk while in the hospital. The majority, 83%, of the women felt that they received enough breast feeding advice and support.

Two-thirds (67%) of the women had a post-natal stay of 3 days or more, and most (80%) felt right about their length of stay. More than half of the respondents (56.7 %) stated that the staff looked after them in a very kind way following the birth of the baby. Less than 10% of women were asked by the HCPs if they had any preference or wanted to follow any particular custom after the baby's birth.

### 3.4. Satisfaction with care

Women's satisfaction with care during pregnancy, labor/birth and after birth in hospital are shown in the Table 4. More than half of the participants were very satisfied with antenatal care (57.5%), intrapartum care (59.2%) and postnatal care in the hospital (59.2%). Nearly one third were somewhat satisfied with the care received during antenatal (35.8%), intrapartum (32.5%) and postnatal periods (35.8%). Less than 10% were somewhat dissatisfied or very dissatisfied with the maternity care received during the three phases of pregnancy. After adjusting for age and parity, country of birth and education level were statistically significant independent predictors of satisfaction with care during pregnancy; country of birth and English speaking (collapsed into with difficulty or not at all, well, fluent) were statistically significant independent predictors of satisfaction with care during labor and birth; while English speaking had only borderline significant association with satisfaction with care after birth in the multivariable analysis. The *p*-values of Hosmer Lemeshow statistics for the three models with these variables were 0.305 > 0.05, 0.852 > 0.05, and 0.843 > 0.05 respectively indicating models fit well. The results of the multivariable analysis (Table 5) reflects that HK born Pakistani women were about four times more likely to be less than very satisfied with the antenatal care when compared to Pakistan born women [adjusted odds ratio (AOR) = 4.1, 95% confidence interval (CI) = 1.3–13.0] and those with education of some university or above were almost 3 times less satisfied with the care received (AOR = 2.9, 95% CI = 1.1–7.4) than those with lower education levels. Also HK born women (AOR = 3.7, 95% CI = 1.2–11.1) and women with English speaking ability of well (AOR = 4.0 95% CI = 1.3–12.0) and fluent (AOR = 3.8, 95% CI = 1.1–14.0) were significantly more likely to be less than very satisfied with the intrapartum care in comparison with Pakistan-born women and women with who could not speak English at all or with difficulty. For postnatal care only those who rated their English speaking as “well” had borderline significantly higher odds of being less than very satisfied (AOR = 2.4 95% CI = 1.0–7.7).

**Table 4:** Satisfaction with maternity care services (counts with percentages, *N* = 120).

**Level of satisfaction**	**During pregnancy**	**During labor and birth**	**After birth**
Very satisfied	69 (57.5)	71 (59.2)	71 (59.2)
Somewhat satisfied	43 (35.8)	39 (32.5)	43 (35.8)
Somewhat dissatisfied	7 (5.8)	5 (4.2)	5 (4.2)
Very dissatisfied	1 (0.8)	5 (4.2)	1 (0.8)

**Table 5:** Multivariable analysis showing AOR for less satisfaction with the overall maternity care services.

**Characteristics**	**During pregnancy**		**During labor and birth**		**After birth**	
	**AOR (CI)**	***P-*** **value**	**AOR (CI)**	***P*** **-value**	**AOR (CI)**	***P*** **-value**
**Country of birth**						
Pakistan	1		1		1	
Hong Kong	**4.1 (1.3–13.0)**	**0.01**	**3.7 (1.2–11.1)**	**0.02**	1.7 (0.6**–**4.7)	0.32
**English speaking**						
Not at all or with difficulty	1		1		1	
Well	1.6 (0.6–4.5)	0.37	**4 (1.3–12)**	**0.01**	2.8 (1.0**–**7.7)	0.05
Fluent	1.5 (0.4**–**5.2)	0.48	**3.8 (1.1–14)**	**0.04**	2.4 (0.7**–**8.1)	0.15
**Education level**						
Secondary and less	1		1		1	
Some university or above	**2.9 (1.1–7.4)**	**0.02**	1.2 (0.5**–**3.0)	0.75	1.4 (0.6**–**3.5)	0.44

AOR, Adjusted odds ratio; CI, Confidence interval; P < 0.05, Significant. The bold values represent statistically significant results.

## 4. Discussion

This study found that the majority of the surveyed Pakistani EM women in HK were either very satisfied or somewhat satisfied with their overall maternity care. More than half of the women described the care they received as kind, respectful and well communicated. Women's country of birth, English-speaking ability and level of education were significant factors associated with level of satisfaction during different phases of pregnancy. HK born Pakistani women expressed relatively less satisfaction with care, especially during pregnancy and labor and birth, as compared with Pakistan born women. Women with conversational or fluent English-speaking ability also felt comparatively less satisfied particularly from intrapartum and postnatal care in hospital. Besides, education level had a negative association with satisfaction with care during pregnancy.

Women's relatively increase satisfaction with care in the present study might be related to developments of maternal health services catering for ethnic minority women over the years. The differences in women's satisfaction in association with country of birth can be explained by women's different expectations of maternity health services and their prior experience of healthcare system (30). Pakistan born women may have previous experience with healthcare in Pakistan, which is a low-income country having less resources as compared to HK. Based on this comparison, these women's expectations of care were fulfilled and they conveyed increased satisfaction with the resources and facilities provided by maternity care services in HK. Similar findings were reported in an Australian study of maternity care experience of immigrant Afghan women in Melbourne, Australia (31). On the contrary, HK born Pakistani women were acquainted with the health services in HK or had past experience of maternity care in HK; as a result, they may have developed greater expectations of care and thus were likely to be critical of the care they received. Similar views were expressed by the UK healthcare professionals providing care to the UK-born EM women. They found UK-born EM mothers to be more self-confident and expressive, and involved in care-related decision making. They had an enhanced understanding of the healthcare system, available resources and expectations of care (32).

Similar to previous research (33, 34), women's expectations and satisfaction with healthcare services also varied with the education level achieved. Women with higher education level tended to be more vocal, information seeking and might anticipate higher level of responsiveness from the healthcare providers. This may explain why Pakistani women with higher education in Hong Kong had less satisfaction with the antenatal services provided.

Effective communication between healthcare professionals and patients is vital for quality care (35); previous research showed language barriers clearly impede good communication between the ethnic minority women and their care givers (12, 36). Yet the settings and findings of the current study are different compared to previous research, which was mainly conducted in English speaking countries (20, 21). In Hong Kong Cantonese is in fact the native language, while English is the second official language (37); in this context, Pakistani women with self-rated conversational or fluent English-speaking ability perceived more language barriers compared to those who had low or no proficiency at all, particularly during labor and birth. The results of this study indicate that most of the women who had no or low English proficiency were provided with interpreting services; as a result, they felt communication was effective and the care received satisfactory. On the other hand, women with increased English-speaking fluency faced difficulties in communication due to the non-availability of English-speaking healthcare professionals, especially midwives and lower-level staff. Therefore, these women felt less responded to and engaged by the maternity care staff and were eventually less satisfied with the care received. A systematic review of immigrant women maternity care experience also highlights the importance of shared language between the women and the service provider. One of the main barriers between immigrant women and their caregivers is the language difficulty which creates hindrance in good communication and understanding. This problem mainly arises when the women are not fluent in the language of the receiving country (29).

The relationship between women and healthcare professionals plays a key role in shaping their perceptions of care, accessing services, utilization and outcomes (12). Although women in this study had increased overall satisfaction with their care, yet their desire for support, kind behavior of staff, understanding and respectful attitude were not fulfilled to a larger extent particularly during labor and birth. Conversely, positive experiences were reported when the staff showed empathy, attended to their individual needs with patience, sensitivity and in a timely manner. These findings are consistent with other studies of ethnic minority women's experience of maternity care (12, 21, 38, 39).

### 4.1. Strengths and limitations

This study has some limitations. Firstly, it did not include the local population for comparison and also the results cannot be implied to other ethnic minority groups. Secondly women who had given birth within the previous 3 years were included in the study, they might have had difficulties in recalling their experiences and introduce potential recall bias. Thirdly our sample size was relatively small as we were not able to achieve our calculated sample size.

Despite of the abovementioned limitations, this study has some strengths too. It is the first to explore the maternity care experience of ethnic minority Pakistani women in HK. Estimating satisfaction is not easy. Respondents may be hesitant to acknowledge dissatisfaction. In this study the interviews conducted by an interviewer of same gender and ethnic group as the respondent, and in the language of women's choice, is probably a protection against the likelihood that dissatisfaction would not be expressed. Involvement of the bilingual researcher sharing the similar ethnicity and cultural upbringing as that of respondents ensured the participation of this hard to reach group. This improves the integrity of the study (40), and indeed the sample was heterogeneous ranging from only Urdu speaking mothers to mothers having English fluency and women who bridged educational spectrum from illiteracy to higher degrees.

### 4.2. Implications

The study demonstrated that effective communication and care that can meet individual expectations, needs and values are essential for providing good quality care to the Pakistani EM women. Maternity care providers should take into account the diversity of EM population in HK. In addition to the provision of interpreters and social assistance to the women with decrease language proficiency and education level, it is also necessary to provide opportunities for the educated and well-spoken women. There is a need to increase the number of Midwives and lower staff with enhanced English language fluency in HK so that language barrier can be overcome and better communication can facilitate better maternity care experience and improve maternal and health outcomes. Thus, it reiterates the importance of provision of person-centered maternity care in improving quality and reducing inequalities among ethnic minority and migrant women.

## 5. Conclusion

This study gives an insight into the evaluation of maternal healthcare system of HK from a sub-group of EM women perspective. Providing person-centered care and addressing barriers in communication can lead to relatively higher level of satisfaction and improvements in the quality of care provided to EM population. Further research is required in this under researched area with the involvement of broader and diverse ethnic minority groups.

## Data availability statement

The original contributions presented in the study are included in the article/supplementary material, further inquiries can be directed to the corresponding author.

## Ethics statement

The studies involving human participants were reviewed and approved by Survey and Behavioral Research Ethics Committee, The Chinese University of Hong Kong, (SBRE-19-521). The patients/participants provided their verbal informed consent to participate in this study.

## Author contributions

Conceptualization and methodology: SA, EN, WG, and DD. Data collection and writing—original draft preparation: SA. Data analysis: SA and WG. Data curation: SA, EN, and WG. Visualization: DD. Writing—review and editing: EN, DD, WG, and E-KY. All authors have read and agreed to the published version of the manuscript.

## References

[1] Millennium Development Goals (MDGs). Available online at: https://www.who.int/news-room/fact-sheets/detail/millennium-development-goals-(mdgs) (accessed January 27, 2023).

[2] Health - United Nations Sustainable Development. Available online at: https://www.un.org/sustainabledevelopment/health/ (accessed August 1, 2022).

[3] Survive Thrive Transform the Global Strategy for Women's Children's and Adolescents' Health (2016-2030) at a Glance. Available online at: www.everywomaneverychild.org (accessed August 1, 2022).

[4] Fair F Raben L Watson H Vivilaki V van den Muijsenbergh M Soltani H. Migrant women's experiences of pregnancy, childbirth and maternity care in European countries: a systematic review. PLoS ONE. (2020) 15:e0228378. 10.1371/journal.pone.0228378 32045416 PMC7012401

[5] Grobman WA Bailit JL Rice MM Wapner RJ Reddy UM Varner MW. Racial and ethnic disparities in maternal morbidity and obstetric care. Obst Gynecol. (2015) 125:1460. 10.1097/AOG.0000000000000735 26000518 PMC4443856

[6] Henderson J Gao H Redshaw M. Experiencing maternity care: the care received and perceptions of women from different ethnic groups. BMC Preg Childbirth. (2013) 13:1–14. 10.1186/1471-2393-13-196 24148317 PMC3854085

[7] Gennaro S O'Connor C McKay EA Gibeau A Aviles M Hoying J. Perinatal anxiety and depression in minority women. MCN Am J Mater Child Nurs. (2020) 45:138. 10.1097/NMC.0000000000000611 31977497 PMC8011863

[8] Shakeel N Eberhard-Gran M Sletner L Slinning K Martinsen EW Holme I. A prospective cohort study of depression in pregnancy, prevalence and risk factors in a multi-ethnic population. BMC Preg Childbirth. (2015) 15:1–11. 10.1186/s12884-014-0420-0 25616717 PMC4310137

[9] Wang E Glazer KB Sofaer S Balbierz A Howell EA. Racial and ethnic disparities in severe maternal morbidity: a qualitative study of women's experiences of peripartum care. Women's Health Issues. (2021) 31:75–81. 10.1016/j.whi.2020.09.002 33069559 PMC7769930

[10] Puthussery S. Perinatal outcomes among migrant mothers in the United Kingdom: is it a matter of biology, behaviour, policy, social determinants or access to health care? Best Pract Res Clin Obstet Gynaecol. (2016) 32:39–49. 10.1016/j.bpobgyn.2015.09.003 26527304

[11] Crowe R. Factors contributing to maternal health inequalities for women who are not white British in the UK. Br J Midwifery. (2022) 30:160–71. 10.12968/bjom.2022.30.3.160

[12] Higginbottom GMA Evans C Morgan M Bharj KK Eldridge J Hussain B. Experience of and access to maternity care in the UK by immigrant women: a narrative synthesis systematic review. BMJ Open. (2019) 9:e029478. 10.1136/bmjopen-2019-029478 31892643 PMC6955508

[13] Crabtree S Wong H. 'Ah Cha'! The racial discrimination of Pakistani minority communities in Hong Kong: an analysis of multiple, intersecting oppressions. Soc Work. 43:945–63. 10.1093/bjsw/bcs026

[14] O'Connor P. Islam in Hong Kong: Muslims and Everyday Life in China's World City. Islam in Hong Kong: Muslims and Everyday Life in China's World City. Oxford: Oxford academic (2012). p. 1–217.

[15] Chen C Chan KH. Type 2 Diabetes Management in Hong Kong Ethnic Minorities: What Primary Care Physicians Need to Know. (2014) 20:222–8. Available online at: https://pdfs.semanticscholar.org/ff2b/9e7316bd519d4611c7f74409d15d998b5da4.pdf (accessed August 1, 2022). 10.12809/hkmj134035 24493686

[16] Vandan N Wong JYH Gong WJ Yip PSF Fong DYT. Health system responsiveness in Hong Kong: a comparison between South Asian and Chinese patients' experiences. Public Health. (2020) 182:81–7. 10.1016/j.puhe.2020.01.019 32200074

[17] Vandan N Wong JYH Fong DYT. Accessing health care: experiences of South Asian ethnic minority women in Hong Kong. Nurs Health Sci. (2019) 21:93–101. 10.1111/nhs.12564 30156370

[18] Garcia R Ali N Guppy A Griffiths M Randhawa G. Ethnic differences in risk factors for adverse birth outcomes between Pakistani, Bangladeshi, and White British mothers. J Adv Nurs. (2020) 76:174–82. 10.1111/jan.14209 31566783

[19] Garcia R Ali N Griffiths M Randhawa G. Understanding the consumption of folic acid during preconception, among Pakistani, Bangladeshi and white British mothers in Luton, UK: a qualitative study. BMC Preg Childbirth. (2018) 18:1–9. 10.1186/s12884-018-1884-0 29902973 PMC6003022

[20] Cross-Sudworth F Williams A Herron-Marx S. Maternity services in multi-cultural Britain: using Q methodology to explore the views of first- and second-generation women of Pakistani origin. Midwifery. (2011) 27:458–68. 10.1016/j.midw.2010.03.001 21036439

[21] Pakistani Muslim Women Birthing in Northern England: Exploration of Experiences and Context - ProQuest. >Available online at: https://www.proquest.com/openview/dbabab0c72b92c9e2607ac14a0f615ae/1?pq-origsite=gscholar&cbl=2026366 (accessed August 1, 2022).

[22] Vangen S Stoltenberg C Schei B. Ethnicity & Health Ethnicity use of obstetrical analgesia: Do Pakistani women receive inadequate pain relief in labour? (2010). Available online at: 10.1080/13557858.1996.9961783 (accessed August 1, 2022). 9395560

[23] Centre for Health Protection - Major Health Indicators in 2020 2021. Available online at: https://www.chp.gov.hk/en/statistics/data/10/27/110.html (accessed August 1, 2022).

[24] Tunçalp Were WM Maclennan C Oladapo OT Gülmezoglu AM Bahl R. Quality of care for pregnant women and newborns - The WHO vision. BJOG. (2015) 122:1045–9. 10.1111/1471-0528.13451 25929823 PMC5029576

[25] World Health. Standards for improving quality of maternal and newborn care in health facilities. WHO. (2016) 2021:84. Available online at: https://cdn.who.int/media/docs/default-source/mca-documents/qoc/quality-of-care/standards-for-improving-quality-of-maternal-and-newborn-care-in-health-facilities.pdf

[26] Lwanga SK Lemeshow S. Sample Size determination in Health Studies. Geneva: WHO (1991). p. 1–88.

[27] Duff LA Lamping DL Ahmed LB. Evaluating satisfaction with maternity care in women from minority ethnic communities: development and validation of a Sylheti questionnaire. Int J Qual Health Care. (2001) 13:215–30. 10.1093/intqhc/13.3.215 11476146

[28] Hennegan J Redshaw M Miller Y. Born in another country: women's experience of labour and birth in Queensland, Australia. Women Birth. (2014) 27:91–7. 10.1016/j.wombi.2014.02.002 24613218

[29] Small R Roth C Raval M Shafiei T Korfker D Heaman M. Immigrant and non-immigrant women's experiences of maternity care: a systematic and comparative review of studies in five countries. BMC Preg Childbirth. (2014) 14:1–17. 10.1186/1471-2393-14-152 24773762 PMC4108006

[30] Lakin K Kane S. Peoples' expectations of healthcare: a conceptual review and proposed analytical framework. Soc Sci Med. (2022) 292:114636. 10.1016/j.socscimed.2021.114636 34894457

[31] Shafiei T Small R McLachlan H. Women's views and experiences of maternity care: A study of immigrant Afghan women in Melbourne, Australia. Midwifery. (2012) 28:198–203. 10.1016/j.midw.2011.02.008 21458892

[32] Puthussery S Twamley K Harding S Mirsky J Baron M Macfarlane A. “They're more like ordinary stroppy British women”: attitudes and expectations of maternity care professionals to UK-born ethnic minority women. Sage J. (2008) 13:195–201. 10.1258/jhsrp.2008.007153 18806176

[33] Chemir F Alemseged F Workneh D. Satisfaction with focused antenatal care service and associated factors among pregnant women attending focused antenatal care at health centers in Jimma town, Jimma zone, South West Ethiopia; A facility based cross-sectional study triangulated with qualit. BMC Res Notes. (2014) 7:164. 10.1186/1756-0500-7-164 24646407 PMC3994781

[34] Quintana JM González N Bilbao A Aizpuru F Escobar A Esteban C. Predictors of patient satisfaction with hospital health care. BMC Health Ser Res. (2006) 6:102. 10.1186/1472-6963-6-102 16914046 PMC1579213

[35] Binder P Borné Y Johnsdotter S Essén B. Shared language is essential: Communication in a multiethnic obstetric care setting. J Health Commun. (2012) 17:1171–86. 10.1080/10810730.2012.665421 22703624

[36] Attanasio L Kozhimannil KB. Patient-reported communication quality and perceived discrimination in maternity care. Med Care. (2015) 53:863–71. 10.1097/MLR.0000000000000411 26340663 PMC4570858

[37] Language Log ≫ Cantonese: Still the Main Spoken Language of Hong Kong. Available online at: https://languagelog.ldc.upenn.edu/nll/?p=33511 (accessed December 21, 2022).

[38] Jomeen J Redshaw M. Ethnic minority women's experience of maternity services in England. Ethn Health. (2013) 18:280–96. 10.1080/13557858.2012.730608 23039872

[39] Redshaw M Heikkilä K. Ethnic differences in women's worries about labour and birth. Ethn Health. (2011) 16:213–23. 10.1080/13557858.2011.561302 21500115

[40] Eboh WO Pitchforth E Teijlingen E van. Lost words: research via translation. Midwives Magazine. (2007). 10:374–8. Available online at: https://go.gale.com/ps/i.do?p=AONE&sw=w&issn=14792915&v=2.1&it=r&id=GALE%7CA169595679&sid=googleScholar&linkaccess=fulltext (accessed December 21, 2022). 17907723

